# Multiple sclerosis and mental health related quality of life: The role of defense mechanisms, defense styles and family environment

**DOI:** 10.3934/Neuroscience.2023027

**Published:** 2023-11-27

**Authors:** Anthi Amaslidou, Ioanna Ierodiakonou-Benou, Christos Bakirtzis, Ioannis Nikolaidis, Theano Tatsi, Nikolaos Grigoriadis, Ioannis Nimatoudis

**Affiliations:** 1 3^rd^ Department of Psychiatry, School of Medicine, Aristotle University of Thessaloniki, AHEPA Hospital, Thessaloniki, Greece; 2 2^nd^ Department of Neurology, MS Center, School of Medicine, Aristotle University of Thessaloniki, AHEPA Hospital, Thessaloniki, Greece

**Keywords:** multiple sclerosis, defense mechanisms, family relations, quality of life

## Abstract

**Background:**

Multiple sclerosis is a demyelinating chronic neurologic disease that can lead to disability and thus to deterioration of quality of life. Psychological parameters such as ego defense mechanisms, defense styles and family environment are important factors in the adaptation process, and as such they can play important roles in QoL. This study aims to assess the psychological factors as well as the clinical and demographic characteristics related to mental health quality of life (MHQoL).

**Methods:**

This was an observational, cross-sectional study conducted in a sample of 90 people with MS in the years 2018–2020. All participants completed the following questionnaires: MSQoL-54, DSQ-88, LSI, FES-R, SOC, BDI-II, STAI. Disability was assessed using EDSS.

**Results:**

In multiple linear regression, significant roles were played by depression (R^2^: 41.1%, p: 0.001) and, to a lesser extent, the event of a relapse (R^2^: 3.5%, p: 0.005), expressiveness (R^2^: 3.6%, p < 0.05) and image distortion style (R^2^: 4.5%, p: 0.032). After performing a hierarchical-stepwise analysis (excluding depression), the important factors were maladaptive defense style (R^2^: 23.7%, p: 0.002), the event of relapse (R^2^: 8.1%, p < 0.001), expressiveness (R^2^: 5.5%, p: 0.004) and self-sacrificing defense style (R^2^: 2.4%, p: 0.071).

**Conclusion:**

Psychological factors play important roles in MHQoL of people with multiple sclerosis. Thus, neurologists should integrate in their practice an assessment by mental health specialists. Moreover, targeted psychotherapeutic interventions could be planned i to improve QoL.

## 1. Introduction

Multiple sclerosis (MS) is a chronic demyelinating disease of the central nervous system, with a variety of manifestations and uncertain course [1]–[3]. It is the most common cause of nontraumatic inflammatory diseases of the central nervous system (CNS), mostly in young and middle-aged adults [1]–[3]. Worldwide, it is estimated that there are 2,800,000 people with MS (PwMS) [4], while in Greece there are about 21,000 [5].

Nowadays, health is being viewed through the scope of a bio-psycho-social model, an aspect of which is to acknowledge that quality of life (QoL) is an important factor in a person's subjective perception of health. According to the World Health Organization, quality of life (QoL) is defined as “an individual's perception of their position in life in the context of the culture and value systems in which they live and in relation to their goals, expectations, standards and concerns” [6]. The term “health-related quality of life” (HRQoL) describes QoL in reference to health problems. Although there is not a universally accepted definition for HRQoL, it refers to a multidimensional, complex concept that includes patient reported outcomes in physical, role functioning, social and psychological aspects of well-being and functioning [7]. A common division of HRQoL is into mental-health related and physical-health related QoL (MHQoL and PHQoL) [8].

MS negatively affects many aspects of the lives of PwMS, physically and mentally, resulting in deterioration of HRQoL [9]–[14]. Many factors are considered responsible, such as demographic characteristics [13],[15]–[17], disease parameters (age of onset, years of disease, disability status, relapse rate, fatigue) [17]–[19], mental health problems [17],[20],[21] and lack of social support [22].

Since QoL reflects in a way the adjustment ability of an individual in difficult situations, we should search among the factors that affect it, the probable psychological adaptive mechanisms. Many personality variables have been linked to HRQoL in PwMS [23]–[28], but there is almost no research regarding ego defense mechanisms, while their impact in HRQoL in other chronic diseases is certain [29]–[32]. The term “defense” was introduced by Sigmund Freud [33] and can be defined as “unconscious psychological responses that protect people from feelings of anxiety, threats to self-esteem, and things that they don't want to think about or deal with” [34]. In this context, a chronic disease such as MS can be viewed as a negative external stimulus which the person has to confront, and defense mechanisms can serve an adaptive role as psychological coping mechanisms. Researchers in psychoanalysis have described several defense mechanisms such as denial, regression, repression, projection, intellectualization, displacement, humor, idealization, acting out, reaction formation, etc. Every person employs a variety of defense mechanisms, different according to different occasions and more or less adaptive [35].

Vaillant, an American psychiatrist and psychoanalyst, studied the adaptive role of defense mechanisms and organized them into four categories, according to the level of maturity that the ego achieves. He named those categories narcissistic, mature, immature and neurotic defenses. This classification is widely used, especially among researchers [36]–[38] (Table 1).

Furthermore, Bond et al. [39], based on this categorization, developed a self-administered instrument, the Defense Style Questionnaire (DSQ), to examine which defense mechanisms are being used by the examinee. Bond differentiated Vaillant's categories a little and introduced the term “defense style” to describe the empirically validated clusters of defense mechanisms that his research brought to view. The defense styles are, in a developmental continuum, maladaptive defense style, image-distortion defense style, self-sacrificing defense style and adaptive defense style [39].

In brief, adaptive defense style allows the person to respond to stress in a positive and creative manner, using humor and functional distraction from distress. The self-sacrificing style includes defenses that reflect a need to perceive oneself as kind, helpful to others and never angry, and those who use them tend to deny their own needs. The image-distorting style includes defenses that alter the perception of oneself and others to either good or bad, strong, or weak, etc., without creating an integrated image, thus avoiding a reality that might be too stressful. Lastly, the maladaptive style includes several defenses that are action-oriented. Those defenses are linked to greater impulsiveness and self or hetero-destructive behavior as responses to stressors [39] (Table 2).

**Table 1.** Vaillant's categorization of defense mechanisms.

**Narcissistic**	**Immature**	**Neurotic**	**Mature**
Psychotic denial Delusional projection	Acting out Fantasy Passive aggression Projection	Displacement Dissociation Intellectualisation Reaction formation Repression	Altruism Anticipation Humor Sublimation Suppression

**Table 2.** Bond's defense styles.

**Maladaptive**	**Image-distortion**	**Self-sacrifice**	**Adaptive**
Acting out Fantasy Isolation Psychotic denial Passive aggression Projection Regression Repression Somatization	Idealization Omnipotence Splitting	Pseudo-altruism Reaction formation	Affiliation Humor Sublimation Suppression

In the current literature, mature defenses are linked to better patient outcomes in several diseases, greater mental health, better patient-doctor collaboration [40],[41], fewer relapses and even greater survival prognosis in cancer patients [42]. Defenses can help the individual cope with the disease stressor in many ways: They can help alleviate the distress [43] (e.g., humor, denial), and mature defenses can help create meaning and perspective for the disease (e.g., altruism, humor), help with connecting and better communicating with others (humor, affiliation) and even translate the distressing experience into a creative project through sublimation [44].

Defense mechanisms can impact an individual's health indirectly, by affecting mental health, interpersonal relations, the doctor-patient relationship, adherence to medication and medical instructions, etc. Thus, mature mechanisms protect people from mental diseases, promote extroversion and empathy and help maintain better relationships and social support. These factors seem to result in better health outcomes [36],[45].

Many studies conducted in different patient samples reveal the importance of mature defenses and adaptive defense style in promotion of health. More specifically, the literature shows that people suffering from chronic diseases are more likely to use less mature defenses or maladaptive defense styles, compared to healthy individuals [29],[30],[46]–[55]. Concerning PwMS, there are only two studies in this domain, which reveal that PwMS more often use immature or neurotic defense styles than healthy individuals [56],[57].

HRQoL can be viewed as a context in which the very process of adaptation is being reflected [58]. Thus, examining the adaptation of an individual that is ill can help evaluate their HRQoL and vice versa. As a result, ego defense mechanisms and defense styles should play a significant role in HRQoL. There are, indeed, several studies that highlight the significance of this relationship and show that the more mature the employed defenses are, the greater HRQoL is [32],[59]–[62]. There is only one study revealing the linkage between poorer HRQoL and immature defense style in PwMS [30].

Another theory that is useful in understanding the coping abilities of an individual is salutogenesis, along with the concept of sense of coherence, introduced by Antonovsky [63]. The term “sense of coherence” describes a health-promoting (in contrast to pathogenesis) concept that enhances resilience to stressful events. Salutogenesis views difficulties as events with meaning, that are manageable, understandable, and predictable, so sense of coherence consists of three components: meaningfulness, manageability and comprehensibility Antonovsky defined sense of coherence as a global orientation that expresses the extent to which one has a pervasive, enduring though dynamic feeling of confidence that (1) the stimuli deriving from one's internal and external environments in the course of living are structured, predictable and explicable; (2) resources are available to one to meet the demands posed by these stimuli; and (3) these demands are challenges, worthy of investment and engagement [64]. As a result of great sense of coherence, the person engages in more positive behavior to cope with stressful difficulties (Antonovsky, 1985). Adopting such an attitude toward disease can help the individual relate more positively to difficulties while maintaining a better psychological state, can result in greater HRQoL [63]. Such an attitude towards a disease can help the individual relate more positively to the difficulties while maintaining a better psychological state, cope in more functional and useful way, and adapt better in the situation, which then can result in greater HRQoL [65]. Research in this field concerning HRQoL and sense of coherence is abundant and validates theory: The greater the sense of coherence is, the greater the HRQoL [65],[66]. The studies referring to PwMS also reveal that sense of coherence is an important factor that enhances HRQoL [28],[67]–[69]

From another perspective, according to Systems Theory, each disease that appears in the family system reflects relational difficulties and should be considered as pathology of the system [70]. Psychological development is absolutely linked to the environment where the individual grows up, and in most cases, this is a family. Life in the family formulates one's character and coping abilities and influences the way the person comprehends how to relate to difficulties. There are several studies that link the course of certain chronic diseases (especially diabetes, cardio-vascular diseases, and arthritis in adults [71]–[75]; and diabetes, asthma, and obesity in children [71],[76]) with social support and family environment. It seems that better communication and cohesion between family members can lead to greater health status or better disease outcomes, while a pressuring and stressful family environment, which exhibits great conflict or control, may result in the appearance of diseases, and affect their course [71]–[76]. There is little research concerning the impact of family environment in HRQoL [76]–[78], and only one study concerning PwMS, which shows that family cohesion and expression are related to better adaptation to the disease [79].

Moos and Moos created the Family Environment Scale (FES) to depict family relations and way of function. The questionnaire is divided into three conceptual domains and evaluates 10 aspects of familiar life [80] (Table 3).

**Table 3.** Family Environment Scale subcategories. https://en.wikipedia.org/wiki/Family_Environment_Scale

Dimensions		
Relationship Dimensions	Cohesion	the degree of commitment and support family members provide for one another
	Expressiveness	the extent to which family members are encouraged to express their feelings directly
	Conflict	the amount of openly expressed anger and conflict among family members
Personal Growth Dimensions	Independence	the extent to which family members are assertive, self-sufficient and make their own decisions
	Achievement Orientation	reflects how much activities are cast into an achievement oriented or competitive framework
	Intellectual Cultural Orientation	the level of interest in political, intellectual and cultural activities
	Active recreational Orientation	the amount of participation in social and recreational activities
	Moral-religious Emphasis	the emphasis on ethical and religious issues and values
System Maintenance Dimensions	Organization	the amount of planning that is put into family activities and responsibilities
	Control	how much set rules and procedures are used to run family life

This study focused on the subcategory of HQoL mental health related quality of life (MHQoL), and the aim was to examine various aspects that might affect it. The main hypothesis was that mature defense mechanisms, adaptive defense styles and greater sense of coherence may promote MHQoL of PwMS. Moreover, we assumed that a family environment that encourages healthy expression of needs and sentiments and allows self-growth would result in greater MHQoL. Considering the abundance of literature in the field of HQoL of PwMS but the lack of studies in the areas of personality factors, this research aspires to make some linkages between MHQoL and defenses as well as familiar factors. Moreover, keeping in mind that various other parameters (such as mental health status and demographic and disease characteristics) play key roles in MHQoL, we hypothesized that the psychological factors mentioned above might be equally important to them. The last step of our analysis was to hierarchize the most important parameters.

## 2. Materials and methods

This is an observational, cross-sectional study conducted in a sample of 90 PwMS, recruited from the Multiple Sclerosis Center of the 2nd Department of Neurology, Aristotle University of Thessaloniki, in the years between 2018 and 2020. The study was designed according to the Declaration of Helsinki and obtained approval from the Bioethics Committee of Aristotle University of Thessaloniki. All participants signed a written consent paper prior to their inclusion to the study.

Inclusion criteria were the definite diagnosis of MS, according to revised 2017 McDonald criteria [81]. Exclusion criteria were age below 18 years, severe cognitive impairment as expressed in lack of concentration or apprehension, poor understanding of Greek language, patients with great disability that were incapable of completing the questionnaires (i.e., difficulty in writing or holding a pen, individuals with tremor or EDSS > 8), patients under psychiatric medication, patients in relapse and, finally, patients that suffered from another chronic disease.

### 2.1. Measures

1) Data referring to the demographic characteristics of the participants and the clinical aspects of their disease (sex, age, family status, number of children, occupational status, disease duration and the event of relapse during the last year) were collected through a questionnaire.

2) EDSS (Expanded Disability Status Scale) [82]. Disability status was determined by a neurologist using EDSS. EDSS is graded from 0 to 10 with increments of 0.5, while 0 stands for no disability, and 10 represents death attributed to MS.

3) MSQoL-54 (Multiple Sclerosis Quality of Life-54) [83]. This is a self-report test, based on SF-36 enriched with multiple sclerosis specific questions. It consists of 54 items, on a Likert scale, and assesses PHQoL, MHQoL and HRQoL as a total, through 12 subscales. The score ranges from 0 to 100 for each category. The scope of this research was to explore probable factors affecting MHQoL, so we used only the MHQoL subcategory. The questionnaire has shown good reliability and internal consistency (Cronbach's α 0.794 in MHQoL).

4) DSQ-88 (Defense Style Questionnaire-88), Greek version [84]. This is a self-report test, consisting of 88 items that examine 25 defense mechanisms [85]. Each item is scored through a Likert scale from 0 (total disagreement) to 9 (full agreement). Defense mechanisms are then categorized into 4 defense styles: maladaptive style, image-distorting style, self-sacrificing style, and adaptive style [23]. Each person employs all defense styles to different degrees, so the scoring depicts the defensive profile of the examinee. Higher scores mean higher use of the particular defense style. Internal consistency for adaptive style was unacceptable, while for the rest it was between 0.705 and 0.844.

5) LSI (Life Style Index), Greek version [86]. This is a self-report test, with 97 items answered with a yes or no response. It concludes in 8 defense mechanisms which, in fact, represent groups of defenses: repression, denial, projection, intellectualization, displacement, regression, compensation and reaction formation [87]. The results from the scoring allow the examiner to identify the extent to which each defense is used by the individual. Higher scores depict higher use of the defense (score 0–1). The reliability of the questionnaire and its internal consistency (except for intellectualization, which was 0.44) were medium (Cronbach's α 0.6–0.78).

6) SOC-29 (Sense of Coherence Scale), Greek version [88]. SOC was developed by Antonovsky, based on his theory about the personal factors that promote health (salutogenesis) [62]. It is a self-report test consisting of 29 items, which the participant scores on a Likert scale of 1–7, depending on the degree of agreement with the statement. The score ranges from 29 to 203 points, and the bigger the score is, the higher the sense of coherence that the person has. The scale has great reliability and internal consistency (Cronbach's α 0.910).

7) FES-R form (Family Environment Scale), Greek version [89]. This is a self-report test, which includes 90 items answered by true-false responses, concerning 10 aspects of familiar life (cohesion, expressiveness, conflict, independence, achievements orientation, active recreational orientation, intellectual cultural orientation, emphasis on religion and ethics, organization, and control) [80]. Each aspect is assessed through 9 questions with a “yes or no” answer. Scoring is 0 to 9 for each category, and the higher the score is, the more that aspect characterizes life in the family. Internal consistency (Cronbach's α) was not relatively good, varying from 0.67 to 0.78.

8) BDI-II (Beck Depression Inventory-II), Greek version [90]. This is one of the most used questionnaires for depression assessment. It is a self-report questionnaire with 21 items, graded from 0 to 3 on a Likert scale. There are questions about physical, emotional and cognitive symptoms. Scores can range from 0 to 63, with scores of 14–19 considered mild depression, 20–28 considered moderate depression and 29–63 considered severe depression. Cronbach's α was 0.920.

9) STAI (Spielberger State-Trait Anxiety Inventory), Greek version [91]. Anxiety was assessed using STAI, which examines two different aspects of anxiety: present anxiety that can be induced by the current situation (state anxiety) and anxiety as a personality characteristic (trait anxiety). It is self-reported and consists of 40 questions, 20 of which assess state anxiety, and the rest of them assess trait anxiety. A Likert scale from 1 to 4 is used. Scores range from 20 to 80. A cutoff of 45 points is considered for existence of anxiety. The higher the score is, the higher the level of anxiety of the individual tested. Internal consistency (Cronbach's α) in both categories is 0.920.

### 2.2. Statistical analysis

Data were expressed as mean ± SD for quantitative variables (age of participants, number of children, EDSS score, years of disease) and as percentages for qualitative variables. The Kolmogorov–Smirnov test was utilized for normality analysis of the quantitative variables.

Bivariate analyses were made by using the Student t-test or Mann-Whitney test, one-way ANOVA Kruskal-Wallis test, and Spearman correlation coefficients (scc) to analyze the relation between the MHQoL subscale of the MSQoL-54 questionnaire (which was defined as the outcome variable), and the quantitative, qualitative demographic and clinical characteristics, respectively.

All demographic and clinical variables and questionnaire total scores which presented p-value < 0.05 in bivariate analyses were included in a multiple linear regression model. The enter method was used to determine the most significant independent factors associated with the outcome variable (MHQoL).

All tests were two-sided, while statistical significance was set at p < 0.05. All analyses were carried out using the statistical package SPSS21.00 [92].

**Table 4.** Demographic and clinical characteristics (original table).

	*No*	*%*	*Mean ± SD*
*Gender*		
Female	61	67.8	
Male	29	32.2	
*Age*			37.90±12.60
*Number of children*			1.0 (0-5)
*Education*		
Primary	3	3.3	
Middle	28	31.1	
High	10	11.1	
Higher-University	42	46.7	
MSc- PhD	7	7.8	
*Marital Status*		
Married	53	58.9	
Unmarried	31	34.4	
Divorced	4	4.4	
Widowed	2	2.2	
*Employment Status*		
Unemployed	44	48.9	
Employed	34	37.8	
Dis. allowance	12	13.3	
*Relapse*		
Yes	21	23.3	
No	69	76.7	
*Years of Disease*			11.50±7.60
*EDSS*			2.5

No: number, SD: standard deviation

**Table 5.** Demographical and clinical parameters and MHQoL (original table).

	**MHQoL**			
	Mean	(SD)	SCC	p-value
*Gender*				
Female	67.05	23.08		ns
Male	63.68	19.97		
*Age*			SCC=-0.074	ns
*Number of Children*			SCC= 0.180	<0.0005
*Education*				
Primary /Middle/ High	65.47	23.74		ns
Higher-University	65.40	20.14		
MSc- PhD	72.24	25.39		
*Marital Status*				
Married	64.42	24.09		ns
Unmarried/Divorced/Widowed	68.18	18.89		
*Employment Status*				
Unemployed	64.20	22.96		
Employed	73.12*	19.17		0.014
Dis. allowance	52.12	19.90		
*Relapse*				
Yes	69,68	19,17		0.016
No	53,76	26,71		0.020
*Years of Disease*			SCC= 0.050	ns
*EDSS*			SCC= 0.303	0.004

SD: standard deviation, SCC: Spearman's correlation coefficient, ns: nonsignificant, *: employed compared to those unemployed and receiving disability allowance

**Table 6.** Psychological parameters, anxiety, depression, and bivariate analysis with MHQoL (original table).

	Mean	SD	Bivariate analysis with MHQoL	
			SCC	P-value
*MSQoL-54 -MHQoL*	65.96	22.07	
*Sense of coherence (SOC)*	135.00	26.84	0.472	<0.001
*Beck Depression Index (BDI)*	11.46	8.89	-0.647	<0.001
*STAI-State*	42.41	12.87	-0.423	<0.001
*STAI-Trait*	42.36	12.27	-0.550	<0.001
*DSQ*		
Maladaptive style	3.78	0.99	-0.462	<0.001
Image-distortion style	3.69	1.12	-0.269	0.010
Self-sacrifice style	4.9	9.2	-0.269	0.010
Adaptive style	5.55	1.25	-0.024	ns
*LSI*		
Repression	0.34	0.19	-0.278	0.008
Denial	0.54	0.19	0.177	ns
Projection	0.70	0.22	-0.102	ns
Regression	0.32	0.21	-0.389	<0.001
Reaction formation	0.38	0.27	-0.117	ns
Compensation	0.39	0.23	-0.098	ns
Intellectualization	0.53	0.19	-0.174	ns
Displacement	0.23	0.17	-0.400	<0.001
*FES*		
Cohesion	6.54	1.88	0.169	ns
Expressiveness	5.48	1.97	0.338	0.001
Conflict	2.37	1.98	-0.225	0.033
Independence	6.32	1.42	0.090	ns
Achievements or.	5.57	1.58	-0.088	ns
Intellectual- cultural or.	5.11	1.96	0.096	ns
Active recreation	4.79	2.31	0.064	ns
Religious- Moral emphasis	4.21	2.01	-0.042	ns
Organization	5.67	1.83	0.099	ns
Control	4.41	1.82	-0.016	ns

SD: standard deviation, SCC: Spearman's correlation coefficient, ns: nonsignificant

**Table 7.** Multiple regression of MHQoL (original table).

	*R^2^*	*reference*	*Beta*	*SD*	*Standardized Beta*	p-Value
Employment (dis. allowance)	<0.5%	Employed	-1.65	4.04	-0.04	NS
Relapse (yes)	3.5%	no	-13.38	4.63	-0.26	0.005
EDSS	<0.5%	---	-1.06	1.22	-0.08	NS
BDI	41.4%	---	-0.99	0.29	-0.40	0.001
STAI STATE	<0.5%	---	-0.12	0.18	-0.07	NS
DSQ image-distortion	4.5%		-0.45	0.14	-0.22	0.032
DSQ self-sacrifice	1.6%	---	-0.40	0.22	-0.17	0.071
LSI repression	<0.5%		1.70	10.36	0.01	NS
LSI reaction formation	<0.5%	---	-5.12	7.25	-0.06	NS
LSI regression	<0.5%		-9.02	12.09	-0.09	NS
LSI displacement	<0.5%	---	-5.61	13.04	-0.04	NS
FES expressiveness	3.6%	---	2.55	1.07	0.23	0.020
FES conflict	<0.5%	---	1.26	1.06	0.11	NS

SD: standard deviation, NS: nonsignificant

**Table 8.** Stepwise model of MHQoL with psychological and clinical factors.

		R^2^	R^2^ change	Beta	SD	Standardized Beta	p-value
1^st^ step	constant	23.7%		108.13	8.33		<0.001
	DSQ- maladaptive style		23.7%	-.34	.07	-0.49	<0.001
2^nd^ step	constant	31.7%		110.41	7.95		<0.001
	DSQ- maladaptive style			-.33	.06	-0.47	<0.001
	Relapse		8.1%	-14.76	4.60	-0.28	0.002
3^rd^ step	constant	37.1%		85.66	11.84		<0.001
	DSQ- maladaptive style			-.26	.07	-0.37	<0.001
	Relapse			-19.56	4.77	-0.38	<0.001
	FES-expressiveness		5.5%	3.05	1.11	0.27	0.007
4^th^ step	constant	39.6%		95.28	12.82		<0.001
	DSQ maladaptive style			-.22	.07	-0.32	0.002
	Relapse			-19.39	4.71	-0.37	<0.001
	FES expressiveness			3.24	1.10	0.29	0.004
	DSQ self-sacrifice		2.4%	-.39	.21	-0.16	0.071

SD: standard deviation

## 3. Discussion and Conclusions

Multiple sclerosis is a chronic disease that largely affects patients' lives, and very often the onset period is in the patients' most productive years [1]. MHQoL is a way to depict the difficulty that PwMS experience from the psychological burden of the disease

The prime aim of this study was to assess the probable psychological factors (personal, endopsychic and familiar) that affect MHQoL. More specifically, we examined the roles of defense mechanisms, defense styles, sense of coherence and family environment. Moreover, we included in our research the most studied factors known to have an impact on HQoL of PwMS and tried to investigate which of these factors are the most important.

Our sample consisted mostly of women (67.8%), and almost half of them were higher education graduates (46.7%). Concerning their employment status, 48.9% were unemployed, while 13.3% received disability allowance. Moreover, 58.9% of the patients were married, with a mean of 1 child (range 0–5).

Concerning the clinical characteristics, the mean disease duration of the patients was approximately 12 years, and three quarters had no relapse in the current year, while the median EDSS score was 2.5 (minimum 0, maximum 7). The average score for patients' MHQoL was 65.96 ± 22.07. Table 4 illustrates the demographic and clinical characteristics of our sample.

Our results concerning the impact of demographic characteristics on MHQoL revealed that only employment status as compared to those receiving disability allowance plays significant role (73.12 ± 19.17 vs. 52.12 ± 19.90, p: 0.014), though in multiple regression analysis this was not considered an important variable. Being employed can, indeed, positively affect MHQoL, according to the literature, mainly because the individual maintains an important social role and feels useful [19]. However, it was an unexpected finding in this research that none of the other demographic factors affected MHQoL, since women, married people, individuals that have completed at least 12 years of education and older PwMS are expected to have higher MHQoL [13],[15],[16].

The analysis of disease parameters affecting MHQoL showed that the event of relapse in the past year and EDSS score (69.68 ± 19.17 and 53.76 ± 26.71, p: 0.016, scc: −0.303, p: 0.004, respectively) had the only important correlations. The negative role of relapse in MHQoL is well established through relevant studies [18],[93]. A relapse might lead to some cognitive and psychological symptoms resulting in worse MHQoL. Moreover, although after a relapse there might not be many or any residual symptoms left, the event of relapse itself reinforces the feeling of uncertainty, stress, and pessimism about the course of the disease, thus deteriorating MHQoL.

On the other hand, since EDSS score reflects the disability status of patients, the greater the score is, the more the person's everyday life is affected. Disability interferes with functionality, and this can change the social roles that someone has and his sense of competence. As a result, strong negative thoughts and feelings may arise, a fact that is depicted in poorer MHQoL [17]–[19]. In the current literature, EDSS has an ambiguous role in MHQoL and has been associated with depression [94],[95], while in our regression analysis disability was not found to be important.

In reference to psychological parameters, sense of coherence is a concept that has drawn the attention of many researchers over the last years, as far as PwMS are concerned. It seems, as confirmed in our own study, that it promotes MHQoL (scc: 0.472, p < 0.001) [28],[67],[68],[96],[97]. Sense of coherence depicts the capacities of an individual to adapt to difficulties and stress. Relating to a negative experience through a way that invests it with meaning and helps apprehend it as manageable is crucial to better psychological adaptation and, in extension, to higher MHQoL. In the multivariate models we were not able to use SOC, due to collinearity with maladaptive defense style. This might mean that those two factors are the counterweights of one another. In fact, maladaptive style is the most immature of all styles and refers to an inability to use internal resources, while SOC indicates the exact opposite state.

The role of certain defense mechanisms in MHQoL is also brought to view in this study. To our knowledge, defense mechanisms have not been previously studied in PwMS. Regression (scc: −0.389, p < 0.001), displacement (scc: −0.400, p < 0.001) and to a lesser extent repression seem to be predictors of worse MHQoL. Regression is a mechanism where the person returns to a previous, less mature psychological and behavioral status, and thus feelings of incapability arise. In this context, the individual perceives themselves as incompetent, dependent on others and vulnerable. This negative sense of self can negatively affect MHQoL. The defense of displacement, according to LSI, refers to the expression of internal distress through explicit aggressiveness. As a result, constant feelings of anger may be present, and this might explain the negative impact in MHQoL. Lastly, the defense of repression helps individuals exclude from their memory unpleasant events but not necessarily the feelings related to them. Consequently, the person can feel puzzled about their real sentiments and even their life story, which can augment distress and deteriorate MHQoL.

In the field of HQoL, overall HQoL of people with chronic obstructive pulmonary disease was found to be negatively impacted by somatization, denial, and undoing [29], and overall HQoL of people with inflammatory bowel disease was deteriorated by somatization and reaction formation [62]. Repression also has a key role, since it seems to deteriorate physical HQoL of breast [98] and colorectal cancer patients [99],[100] and social HQoL of patients with arthritis [101]. We observe that research on HQoL is scarce, the results differ according to patient groups and, most importantly, there is no literature related to MHQoL. This study sheds some light to this aspect of HQoL, although none of the defense mechanisms was found to play significant role in multiple regression analysis.

Regarding the role of defense styles in MHQoL it was revealed that mostly maladaptive defense style (scc: −0.462, p < 0.001) but also image-distorting defense (scc: −0.269, p: 0.10) and self-sacrificing styles (scc: −0.269, p: 0.10) impair MHQoL. Maladaptive defense style plays a major role in MHQoL, a finding which is in accordance with Bond's theory. Using the maladaptive defense style, which is the most immature style, the person deals with emotional conflicts by expressing their feelings against someone or something in an indirect and often self-destructive manner [39]. In fact, maladaptive defense style has been linked to poor coping [102], so the person cannot manage stress, unable to use his internal resources. Defense mechanisms linked to maladaptive style are projection, displacement, passive-aggressiveness, acting out, somatization and fantasy.

Image-distorting defense style refers to the idea of people being good or bad, weak or strong, with no intermediate states. This might be very confusing for a person since someone who was thought to be a great doctor, for example, after a relapse can be viewed as the worst one by the patient [39],[102]. Moreover, lack of confidence in the world can lead to stress, lack of self-confidence and pessimism, while according to Bond image-distorting defense style is linked to instability in the sentiment, behavior and mood [102]. All of the above can lead to poor MHQoL. When the self-sacrificing defense style is implemented, the individual has the tendency to deny their own needs in favor of other people's demands, while they perceive themselves as always kind, available and never angry [39],[102]. This defense style is more closely to adaptive style in a developmental scale. There are only a couple of studies in the domain of HQoL of individuals with chronic diseases and defense styles. Maladaptive style deteriorates environmental HQoL of systemic sclerosis lupus erythematosus patients [32] and physical, mental, and social HQoL of systemic sclerosis patients, while self-sacrificing style impairs environmental HQoL of people with arthritis [30]. To our knowledge there is no literature concerning PwMS.

Another new finding is the role of family environment in MHQoL: High conflict between the members of the family deteriorates MHQoL (scc: −0.225, p < 0.05), while on the contrary, expressiveness enhances it (scc: 0.008, p < 0.005). An explanation could be that when a person lives in a familiar environment where it is encouraged to openly express negative feelings and thoughts without negative judgment from the other members, they feel less rejected and more secure and self-confident. As a result, the person can cope better in external difficulties. On the other hand, constant arguments, aggressiveness, and expression of anger may lead to feelings of insecurity, low self-worth and incompetence, as well as anxiety and sadness. There is some research that links poor psychological adjustment of children with chronic diseases to confronting family relationships and better adjustment to a more adaptive environment [103],[104]. To the best of our knowledge there is no other research in this domain. Few studies that highlight the importance of conflict as a maladaptive factor, they do not refer to the mental component of HQoL, and the study group was a pediatric population [76]–[78]. In multiple regression analysis, conflict did not exhibit an important role as a variable of MHQoL.

The negative role of anxiety in maintaining good MHQoL was highlighted in our research and remains a common finding (STAI State scc: −0.423, p < 0.001, STAI Trait scc: −0.550, p < 0.001) [21],[22],[27],[94],[105]–[110]. In fact, despite being a normal reaction to phobic stimuli, when it is over-activated, anxiety can be detrimental to the way people relate to difficulties. Anxiety can create a more negative framework through which people perceive reality and its consequences. In the case of MS, anxiety is related to the uncertain course of the disease and the stress that occurs concerning future health status. However, surprisingly, anxiety was not found to be a significant factor in multiple regression analysis.

People suffering from depression appear to have lower MHQoL (BDI scc: −0.6470, p < 0.001), a finding shared by many researchers [17],[20],[21],[94],[106]–[110]. Depression is present in 35% of PwMS [107]. Apart from depression being an independent entity, there is also the point of view that the disease itself is responsible for the occurring depressive symptoms, through demyelination in certain brain regions [104]. Nevertheless, the assessment of MHQoL in MSQOL-54 shares common items with BDI-II (assessment of mood, cognitive status, and social function), so there is an immediate relationship between those two elements. After having completed the bivariate analyses, we performed a multiple linear regression analysis, to define which of the factors that were statistically significant in previous analyses affect MHQoL the most (Table 7). STAI-Trait, SOC and maladaptive defense style were excluded from the analysis because of collinearity with BDI and low contribution to the model (R^2^ < 0.5%).

Summarizing the results of the regression analysis, we observe that the independent factors explain 57.4 % of the variability of MHQoL, with depression being the most significant factor of MHQoL (R^2^: 41.1%, beta: −0.99 ± 0.29, p: 0.001). This may reflect the similarities between the clinical characteristics of depression and the components of MHQoL. However, depression itself can be a debilitating factor when it comes to health perceptions, even more since depression alters cognition. As a result, pessimistic point of view and difficulties in attention, concentration, and memory affect negatively MHQoL. Other significant factors were the event of relapse during the previous year (R^2^: 3.5%, beta: −13.38 ± 4.63, p: 0.005), self-sacrificing and image distortion defense styles (R^2^: 4.5%, beta: −0.45 ± 0.14, p: 0.032) and expressiveness in family environment (R^2^: 3.6%, beta: 2.55 ± 0.7, p < 0.05).

Since depression refers to 1/3 of PwMS, we decided to run another analysis excluding it from the model, and we performed a hierarchical-stepwise analysis (Table 8). Depression was excluded because in our sample the depression rates were small, and we wanted to identify which of the other variables had the biggest impact on MHQoL. SOC and self-sacrificing defense style were excluded from the analysis because of collinearity with maladaptive defense style and low contribution to the model (R^2^ < 0.5%).

This time, maladaptive defense style comes up in front as the most important factor affecting MHQoL (R^2^: 23.7%, beta: −0.34 ± 0.07, p: 0.002). The event of relapse (R^2^: 8.1%, beta: −14.76 ± 4.60, p < 0.001), expressiveness (R^2^: 5.5%, beta: 3.05 ± 1.11, p: 0.004), and to a lesser extent, self-sacrificing defense style (R^2^: 2.4%, beta: −0.39 ± 0.21, p: 0.071) also affect MHQoL. In total, these factors explain 39.6% of the variability of MHQoL.

Our results highlight the importance of certain factors in patients perceived psychological adaptation to MS. In this study, we included a great variety of potential factors that could affect MHQoL, and it seems that, apart from the event of a relapse in the past year, MHQoL is basically linked to psychological factors. Even relapse could impair MHQoL through a psychological mechanism: the arousal of negative feelings about physical condition and about the future. The role of depression has already been studied, but defense mechanisms and particularly the role of the family have not been investigated before.

MHQoL is a broad concept that includes many aspects of a patient's experience and self-perception. Our findings validate the importance of a bio-psycho-social model of health and show that a more holistic approach to MHQoL (that includes demographical, clinical, personal, endophytic and social-familiar factors) is necessary.

Moreover, we should emphasize the need for psychiatric and psychological assessment of all PwMS on a routine basis, as psychopathology, on the one hand, and psychological parameters, on the other hand, seem to play crucial roles in MHQoL. It is important that PwMS are referred to a psychiatrist to be assessed and, when needing treatment, for depression. The next step should be a thorough psychological assessment by a mental health specialist to identify the constellation of defense mechanisms and defense styles used by the person. Especially when great use of maladaptive defense style is detected, an intervention is essential, to help the individual use different, more adaptive defense mechanisms. Another substantial step should be the assessment of family relationships and familiar environment. Family members should be referred to a family therapist when the family members maintain a judgmental attitude toward the expression of feelings and thoughts.

Concluding, seeking the personality aspects, as well as the family characteristics, of each of the PwMS individually can provide important information about their adaptation ability and of course personal needs in terms of psychological support. As a result, neurologists can expect better MHQoL outcomes, as the role of psychotherapy in enhancing QoL of PwMS is well established [110].

However, this study has some limitations. First of all, this study was conducted during the period 2018–2021, when the pandemic of Covid-19 occurred. Since this is a new circumstance, an unpredictable and stressful one with many restrictions in everyday life, it is possible that it might have affected not only the way people cope with it but also QoL itself, mentally and physically. Another limitation is the small sample used in this study, which cannot allow us to generalize the results. Further studies need to take place, with larger numbers of participants. Finally, the assessments of MHQoL, psychopathology and psychological parameters were held through self-report measures. It is obvious that no questionnaire can replace the role of a well-trained professional, and thus we suggest that the results be considered with caution.
